# Racialised capitalism, decoloniality and the university: an exploration of the colour line and colonial unreason in higher education

**DOI:** 10.3389/fsoc.2023.979579

**Published:** 2023-09-22

**Authors:** Natalie Tegama

**Affiliations:** Faculty of Wellbeing, Education and Language Studies, The Open University, Milton Keynes, United Kingdom

**Keywords:** racialised capitalism, coloniality, racial amnesia, decolonisation, university, higher education

## Abstract

Over the last decade, there has been an increase in calls to address important questions on race and decolonisation within the university, administratively, pedagogically, and socially. This study investigates the relationship between the university, the coloniser, and the colonised during the colonial era and the afterlife. It aims to demonstrate that the university has made the act of abstraction and theorisation central across disciplines in a way that shears theoretical principles from the historical contexts they emerge from, distancing them from the purposes, people, and interests they were meant to serve, as well as the populations they were meant to dispossess and disempower. The study provides a conceptual framework for deconstructive analysis of the university’s pedagogical operations and societal function with the view to elucidate the university’s colonial and racial blind spots, notably, with a reliance on disciplinary narratives from development, international relations, and international law to offer tentative answers to the questions of decolonial praxis, the decolonial scholar, and coloniality in the contemporary university.

## Growing discontent in higher education

Over the last decade, there has been an increase in calls to address important questions on race and decolonisation within the university, administratively, pedagogically, and socially. Questions have arisen on “persistent racial equality gaps” in higher education (Advance HE, 2021; Thomas and Quinlan, 2021, p. 37). These include questions on institutional bias in admission processes and degree awarding gaps, that is, the proportional difference between the ethnic minority students in the UK and white students who are awarded a first or 2:1 degree classification upon graduation. These awarding gaps have long-term consequences that play out in the social strata over a lifetime, in economic gains and social disadvantages (Thomas and Quinlan, 2021; Wong et al., 2021). The summer of 2020 was marked by international events that brought to the surface issues of systemic racism and questions on the bearing of a colonial past on the present, across many sectors. In higher education, these questions were met by scholars such as Bhambra et al. (2018), who have long been calling for the decolonisation of the university and highlighting shortcomings in the sector such as the glaring lack of diversity across faculties and the exceptionally low numbers of Black female professors across British universities (Rollock, 2021). For ethnic minority students, this moment of collective questioning gave them a platform to voice their stories of (un)belonging and their need for safe spaces on campuses that can be hostile environments for Black and ethnic minorities who rarely see themselves, their cultures, perspectives, and lived and historical truths within lecture rooms and across Eurocentric curricula (Shieber, 2020; Thomas and Quinlan, 2021).

The pertinence of questions on race, decolonisation, equity, and equality where the university is concerned are in part embedded in what happens beyond the university, within the social sphere where the university has historically played an important role as a site of social and scientific innovation (Tegama and Fox, 2023) and as a source of the workforce that contributes to societal outcomes. This study is, therefore, premised on the argument that the perception of the university both internally and externally as either a colonial agent or an innocent bystander is important to understanding whether the university is a dismantler or a producer/reproducer of racial inequities in contemporary society. It is, therefore, my aim to map the relationship between the university, the coloniser, and the colonised and draw lines of continuity in the underpinning logic on which colonialism was built to show the persistence of the same logic in the colonial afterlife within higher education. I aim to demonstrate that the university has made the act of abstraction and theorisation central across disciplines in a way that shears theoretical principles from the historical contexts they emerged from, distancing them from the purposes, people, and interests they were meant to serve, as well as the populations they were meant to dispossess and disempower (Krishna, 2001). I combined contemporary postcolonial/decolonial thought and Black radical thought to map a broad picture of the interplay between theory and practice. I rely on the disciplines of international relations and international law and development to illustrate my argument and offer tentative answers to questions on decolonial praxis and the role of the decolonial scholar and scholarship in subduing coloniality in the contemporary university.

## Making the colour line visible

At the turn of the 20th century, Du Bois conceptualised the problem of the 20th century that has been sustained into the 21st century as that of ‘the colour line’—the question as to

how far differences of race which show themselves chiefly in the colour of the skin and the texture of the hair – [would thereafter] be made the basis of denying to over half the world the right of sharing to utmost ability the opportunities and privileges of modern civilization? (Du Bois, 1900, p. np).

There is a growing body of contemporary postcolonial/decolonial literature that is formulating important questions around the appearance, codification, and recodification of ‘the colour line’. de Sousa Santos (2014) conceptualises it as ‘an abyssal line’—the radical divide that is a product of the imperial project of global colonialism and capitalism—that upholds the systems of inequality that govern our current social realities. Within the university, students and teachers are formulating important questions concerning the purposes, pedagogical functions, and significance of the university in cultural reproduction and societal formulation through pedagogic action. They are mapping the colour line in their classrooms and seeking philosophical plurality on the curricula with the view to decolonise (Shieber, 2020).

‘Decolonisation’ “takes colonialism, empire and racism as its empirical and discursive objects of study” (Bhambra et al., 2018, p. 2). It uses them as points of departure to map how they have shaped the present contemporary context whilst remaining “effaced from view” (Bhambra et al., 2018, p. 2). Decolonial work seeks to ‘make visible’ and ‘make room’. Decolonisation is, therefore, a call to *make visible* the abyssal line that marks the divide between those governed by emancipatory regulation and those governed by legitimated forms of violence—epistemic, economic, and physical that are bound to a colonial past. It is also a call to *make room* for alternative ways of thinking about our world, pulling us away from the Eurocentrism that legitimated colonial violence and moving us toward greater racial equality. There is a necessary plurality in what that may look like. This is because of the multiplicity of spaces that experienced colonial violence and the heterogeneity in the instruments of violence, including the weaponisation of knowledge production about previously colonised people as well as in previously colonised spaces. Ndlovu-Gatsheni (2018, p. 8) contends that “since power and knowledge are inextricably intertwined, control of the domain of knowledge generation and knowledge cultivation remain important for the maintenance of asymmetrical global power structures in place since the dawn of Euro-North American centric modernity.” Since the 19th century, the university, as an institution, has played a key role as a site of knowledge production, as well as scientific and social innovation (Tegama and Fox, 2023). Within the scope of this study, I explore the university as a place of learning that is a powerful instrument for social reproduction.

## Learning and inequality in learning

Sfard (1998) offers useful thinking on the metaphors of learning, which divides language on knowledge into two distinctive schools of thought, namely, the acquisition and participation metaphors. The former encumbers the idea of passive reception of knowledge, and the language around it treats “the human mind as a container to be filled with certain [learning] materials” (Sfard, 1998, p.5). The participation metaphor features language around discourse and communication and views learning as a process of becoming a member of a certain community, learning the language and norms particular to a community, and participating within it (Wenger, 1998). In Sfard’s (1998) conceptualisation, newcomers to the community have the potential to change and reform the community. Learning is an ever-evolving process within which the role of the teacher is to preserve continuity. Sfard’s (1998) two metaphors are not mutually exclusive and can coexist within one learning experience, and some scholars argue them to be necessary (Anderson et al., 1997; Sfard, 1998). If we conceptualise the pedagogy, curriculum, and assessment frameworks of the university to be underpinned by the first of the metaphors, then we might ask questions about what it is that students acquire. If the conceptualisation of learning is underpinned by the latter, then we might ask questions about the norms and rules of the community. In this case, we might ask questions specific to racial inequality in the post-colonial era and decolonisation across the disciplines to explore the normative frameworks that form the foundations and discursive boundaries that guide disciplines.

Scholarly attempts to address questions of racial inequality in the post-colonial period are often bound to contemporary postcolonial and decolonial thought or Black radical thought (Danewid, 2018). Choosing to sit in the contemporary camp is oftentimes defined by a focus on Eurocentrism, as well as cultural and intertextual analyses (de Sousa Santos, 2014; Mignolo and Walsh, 2018; Ndlovu-Gatsheni, 2018). The Black radical thought camp adopts a material lens, taking on a Black/decolonial Marxist approach that focuses on a racialised global political economy and raises questions around materiality, alongside troubling the exploitation that sits at the core of the global system (Robinson, 2000; Ndlovu-Gatsheni and Ndlovu, 2021). My goal in this article is to provide a conceptual framework for deconstructive analysis of the university’s pedagogical operations and societal functions with the view to elucidate the university’s colonial and racial blind spots that are a product of wilful racial amnesia (Krishna, 2001).

## Recognising and erasing the colour line

I join the growing number of scholars who are boundary crossing, marrying both the contemporary and Black radical thought camps, to explore pluriversal approaches to epistemology and global political economy (Danewid, 2018). These include scholars, such as Tzouvala (2020), whose intertextual analysis of international law borrows from Capers (2006) *Reading Back, Reading Black,* an oppositional reading method that looks for tension and deficiency in legal reasoning. This opens the door to examining the underpinning logic to read not only what is there but what is omitted, what is silenced, and who is dispossessed in the silence. Scholars such as Tzouvala (2020) effectively trouble the manner in which the arc of international law bends toward constructing and maintaining racist justifications of global inequality that are intimately tied to Eurocentrist notions of the “restern world” — Ndlovu-Gatsheni and Ndlovu (2021, p. 89), conceptualisation of the rest of the world beyond the West that was subjected to colonialisation and treated as “civilisationally inferior” (Tzouvala, 2020, p. 1).

Tzouvala (2020) in her intertextual analysis of the law makes visible the codification of the colour line in the global political economy by centring materiality and exploring the specific ways that international law uses intellectual tools to ‘objectively’ differentiate between states and articulate the standard of civilisation. For example, ‘objective differentiation’ can be shown through the enactment of indexes for state-based ranking systems that measure state creditworthiness. These ‘objective’ international tools are tied to power and wealth inequalities and by extension the reproduction of systems of domination and economic exploitation of former colonies by imperial metropoles. Power is, therefore, mediated by intellectual tools and the institutions that conceive them. As such, it is important to understand the function of and engage in the critique of the institutions through which power is exercised.

The university as a site of social and scientific innovation constructs, maintains, and evolves intellectual tools through which power is mediated. This necessitates a critique of the university and an examination of its history and engagement in producing iterations of the colour line through the use of intellectual tools (Chomsky and Foucault, 2015). This requires troubling the notions of neutrality and independence in the framing of the university, with the goal of wrestling with how the university ought to be framed and where on a continuum that runs from an innocent bystander to a colonial agent it ought to sit. I contend that this would require a ternary approach to critiquing on multiple levels, which I adopt in this study.

## Taking a ternary approach

In adopting a ternary approach, I initially engage in critiquing at the level of the institution where I critique the function of the institution in the broader sense. I borrow useful thinking on the ideological function of the education system from Bourdieu and Passeron (1990) with the view to frame the university’s relative autonomy and the presence of imperialist ideology. This enables me to examine how imperialist ideology has shaped the philosophical assumptions of particular disciplines (Ndlovu-Gatsheni and Ndlovu, 2021). I then engage in critiquing on two additional levels, looking across and within disciplines. This facilitates the exploration of how disciplines or aspects of disciplines perform functions that dismantle or produce/reproduce racial inequality or form the intellectual tools that enable other disciplines to produce/reproduce racial inequality. Examining disciplines in this way facilitates inquiry into the metamorphosis of the colour line within the context of the university. It empowers historically dispossessed individuals to ask of the university salient questions of culpability and accountability with specific reference to racial injustice and the legacy of a colonial past and its bearing on the contemporary present, both within the university and beyond. This requires the deconstruction of disciplines, looking both within and across disciplines to explore how intellectual tools are used to create and maintain justification principles that are used to establish the rules of governance and academic conventions, as well as how these, in turn, legitimise and uphold injustices that conform to the imperialist ideology that underpins racialised capitalism. These mechanisms, in turn, contribute to structuring our world in a way that reproduces inequality.

I routinely return to the notion of the colour line, legitimacy, and the exertion of power, epistemologically, ideologically, and practically. Racialised capitalism, decoloniality, and the university in contemporary society are explored in contexts and with anecdotal narratives that facilitate an examination of the paradoxes of this post-colonial moment across the university. This is done with a notable reliance on the humanities that make it possible to illustrate the different ways in which coloniality materialises to produce and reproduce colonial patterns that speak of a deference to shifting beyond what I argue to be a race-based moral relativism that is present in the way certain disciplines are taught. The ideas articulated in this study echo in many ways the discontent of decolonial scholars across the globe, whose justice-seeking work centres on epistemic freedom and plurality in knowledge (Mignolo and Walsh, 2018; Ndlovu-Gatsheni and Ndlovu, 2021). I, therefore, borrow from their study in exploring the colour line within higher education in the United Kingdom.

## Contextualising this post-colonial moment

Centralising the question of the colour line in the colonial afterlife necessitates the historical contextualisation of this post-colonial moment in reference to Europe’s modern history of imperialism. Whilst the primary point of reference in this study is the third wave of imperialism in modern European history that culminated in the scramble for Africa in the late 19th century, colonising Africa into the turn of the century such that just over half a century ago, “most of humanity was living under the yoke of colonialism” (Mbembe, 2021, p. 1). To understand the interplay that exists between justification principles, rules of governance, and academic conventions that legitimise unequal power relations between the global north and south, it is important to remain cognizant of the broader background of the three waves of imperialism in modern European history. Dating as far back as the Iberian and Dutch conquests between 1520 and 1620—these would go on to be foundational to our understanding of international governance (I will return to the question of the Dutch in subsequent sections). After the wave of European empires stretching across parts of Asia, America, and Australasia, there followed the colonisation of Africa (Mbembe, 2021).

Situating the post-colonial period against the backdrop of imperial history importantly highlights the infancy of the decolonial project in comparison to the amount of time that imperialism had to destruct, destabilise, and reshape human history. Along with the nuanced ways in which its violent dominance defined social and cultural logic based on the sustained period of imperialist ideology, which has, in part, shaped the university, the assumptions on which disciplines are built and its function in society. This is no more relevant than in the fields of international relations and international law where there are key scholars such as Hugo Grotius, who is often read and taught as an objective founding father of international law, despite his being intimately linked to imperial conquest (Krishna, 2001). For example, his influential work *Mare Liberum* or *The Freedom of the Seas* was effectively a paid deliverable for the Dutch-East India Company, driven by the Dutch’s desire to increase their share of colonial trade (Krishna, 2001). What decolonial scholars, such as Krishna (2001), challenge is the practice of teaching the work of such scholars as value-free, despite the underpinning logic that served to coalesce “an incipient Europe against the “other”—variously defined as the Islamic Middle East, the despotic Asians, the propertyless Indians of the New World, and the slaves of Africa” (Krishna, 2001, p. 209).

The contention is, therefore, against the canonisation of works underpinned by colonial logic and the redistribution of those works in the university as value-free knowledge that assumes a point of neutrality predicated on racial amnesia to distance theories and abstractions from their racist genesis (Krishna, 2001), effectively sanitising the colour line and anchoring changes in its “appearance and codification” (Robinson, 2000, p. 2). Quijano (2000) coined this continuity of colonial logic and the sustenance of its matrices of power through recodification: coloniality. He referred to the ways in which colonial logic manifests in contemporary society, its ordering and hierarchization of race and knowledge to create and normalise a “Eurocentric techno-scientific instrumental rationality” (Ndlovu-Gatsheni and Ndlovu, 2021, p. 90). It is bound to a cycle of production that reproduces racist relations that are defined by domination between the colonised and the coloniser. These are made visible by the extractive and exploitative relations that currently expand beyond the West, i.e., the United States and Europe (Mignolo and Walsh, 2018).

## Contextualising social and cultural logic

Quijano unveiled the concept of coloniality/decoloniality in “the hinging moment on the closing of the Cold War and the opening of the neoliberal global design” (Mignolo and Walsh, 2018, p. 6). The world is reeling from presently experiencing both sides of the neoliberal pendulum, simultaneously facing multiple crises, not least among them are a warming planet, environmental crises, and health security threats (Magnano San Lio et al., 2023), of which many are disproportionately affecting black and brown bodies. This too is a hinging moment, that is, a narrow window to pause and examine this post-colonial moment and the ways in which disciplines and schools of thought that have shaped our current global order and the economies that have failed to nourish and protect an astounding portion of the global population. This highlights the need to engage in the process analysis of the recodification of the colour line and to reimagine planetary governance. To consider what it may mean to “*delink*” the praxis of living from coloniality (Amin, 1987), as well as consider what the role of the academic and the university may be in that process, in order to create room for analysing the disciplinary justification principles that form the “the basis of denying to over half the world the right of sharing to utmost ability the opportunities and privileges of modern civilization” (Du Bois, 1900, p. np). By mapping the disciplinary genesis of justification principles, we can, for example, consider the treatment of labouring black and brown bodies as expendable in global value chains or how the politics of othering and exclusion with links to migration or tourism are perniciously built into academic discourse (Banerjee, 2008; Chambers and Buzinde, 2015; Gradin, 2016). In doing so, there is room to mark interrelated points within and across disciplines to find thematic continuity underpinned by coloniality. This requires us to collapse the borders of disciplines and adopt an interdisciplinary lens that has the capacity to find continuity and associations to the colour line within and across disciplines, linking them to the praxis of living and bringing “insights to bear on what has been for the most part a very straitened, abstract, ahistorical and deeply Eurocentric way of talking about issues such as global inequality, security, migration, trade, refugees, environmental crises, etc.” (Krishna, 2021, p. np).

Embedded within interdisciplinary scholarship is the opportunity to make apparent that theories and concepts do not emerge from or exist in a vacuum that is devoid of social and cultural logic. Instead, they are underpinned by assumptions and driven by arguments that produce and reproduce concepts that are in keeping with cultural logic. The criticism that has reared its head in the context of the university has been that its cultural logic has been broadly based on Eurocentric epistemology and well-crafted systems of oppression that, in their theorisation, desensitise and conceal the crimes of racialised capitalism and coloniality (Ndlovu-Gatsheni and Ndlovu, 2021, p. 1). It is, therefore, important to elucidate the underpinning cultural and social logic to engage in the process analysis that makes apparent the interplay between theory, social mechanisms, and the university’s role in the legitimisation of unequal power relations. In the subsequent sections, I examined the question of legitimacy and the making of social-cultural logic.

## The making of social and cultural logic

The theoretical supposition from which this study emerges borrows from Nescolarde-Selva’s and UsóDoménech’s (2014, p. 63) conceptualisation of “successive… systems of cultural conventions” that play varying roles in carrying out ideological objectives and Beetham’s (1991) theoretical analysis on the legitimation of power. To begin with, I expound on Beetham’s analysis, then discuss and adapt Nescolarde-Selva’s and UsóDoménech’s approach to social-cultural logic in the context of the university, and routinely return to legitimacy to explicate my argument.

## Dominance and subordination

Beetham (1991) asserts that social relations of power are predicated on the degrees of separation that exist between the dominant and subordinate parties, as well as the rules of access and exclusion that govern that separation. Separation is justified based on the possession of certain qualities in the dominant party that are lacking in the subordinate. Those qualities are then treated as commensurate to the power that the dominant party can exercise. In the context of the university and exclusionary Eurocentric debates on who should be on the curriculum, these are fundamentally underpinned by imperial logic and the elusive and ever-changing standard of civilisation that can simply be defined as the colour line, whether the separation is linguistically or empirically coded. The dominant, i.e., Eurocentric scholars’ attempts to legitimise the exclusion of global south scholars from the curriculum are often bound to claims that their works fail to meet the required ‘*standards of civility’,* albeit linguistically coded (Tzouvala, 2020). For example, in philosophy where the debate of exclusionary practice often arises, traditions of philosophy from the global south “are deemed culturally inferior because of the indigeneity of their practitioners…[thus] the rejection or acceptance of any philosophical work/theory/orientation [is subject to] the indigeneity of its authors or cultural influence rather than on the veracity, plausibility, and viability of its proposition” (Chimakonam and Nweke, 2018, p. 279).

Understanding legitimacy requires the identification of multi-dimensional, qualitatively different elements that constitute legitimacy. As such, power can be considered legitimate if.

- it conforms to established rules.
- the rules can be justified by reference to beliefs shared by both the dominant and subordinate.
- There is evidence of consent by the subordinate to the particular power relation” (Beetham, 1991, p. 16).

**Figure 1:**
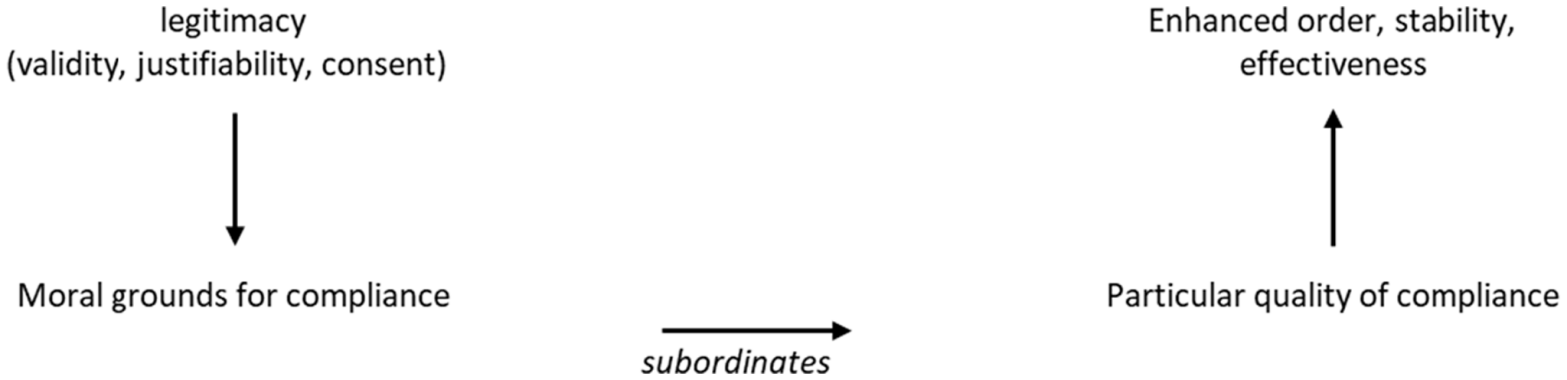
Characteristics of a power system or relationship (Beetham, 1991, p. 34).

These elements then shape the characteristics of a power system. Legitimacy establishes moral grounds for compliance from subordinates, whose quality of compliance affects the order, stability, and effectiveness of the power system. Figure 1 displays the characteristics of a power system, as illustrated by Beetham (1991, p. 34).

To trouble the legitimacy of Eurocentric practice within disciplines in the university and the ‘*grounds for moral compliance’*, I turned my attention to *‘the establishment of the rules’* throughout the three waves of modern European imperialism. I used Nescolarde-Selva and Usó-Doménech’s (2014) conceptualisation of successive systems to demonstrate the genesis of contemporary imperial logic and the process of rebranding imperial logic as shared beliefs for contemporary society.

I highlighted education’s role in rebranding imperialism in the face of declining colonial empires and the rise of antiracist movements that turned the tide and made biologically racist justifications and colonial rhetoric politically unsavoury (Tzouvala, 2020). In turn, I place greater emphasis on the incipient use of intellectual tools of concealment to maintain legitimacy and re-establish moral grounds for compliance with structures that maintained unequal power relations. Throughout the process, the university has evolved its use of language to legitimise coloniality, and the roles of disciplines in managing the colonial have also changed. For example, where the anthropologist was often found at the side of the colonial administrator, the development scholar currently finds themselves at the side of the foreign aid body of the government. Although language has shifted, the study of development is fundamentally underpinned by imperial logic and the colour line, linguistically coded as ‘developing countries (Du Bois, 1900; Tzouvala, 2020). I will return to the matter of disciplines in subsequent sections. In this section, I refocused my attention to the making of social and cultural logic.

## Superstructures and epistemic norms

**Figure 2:**
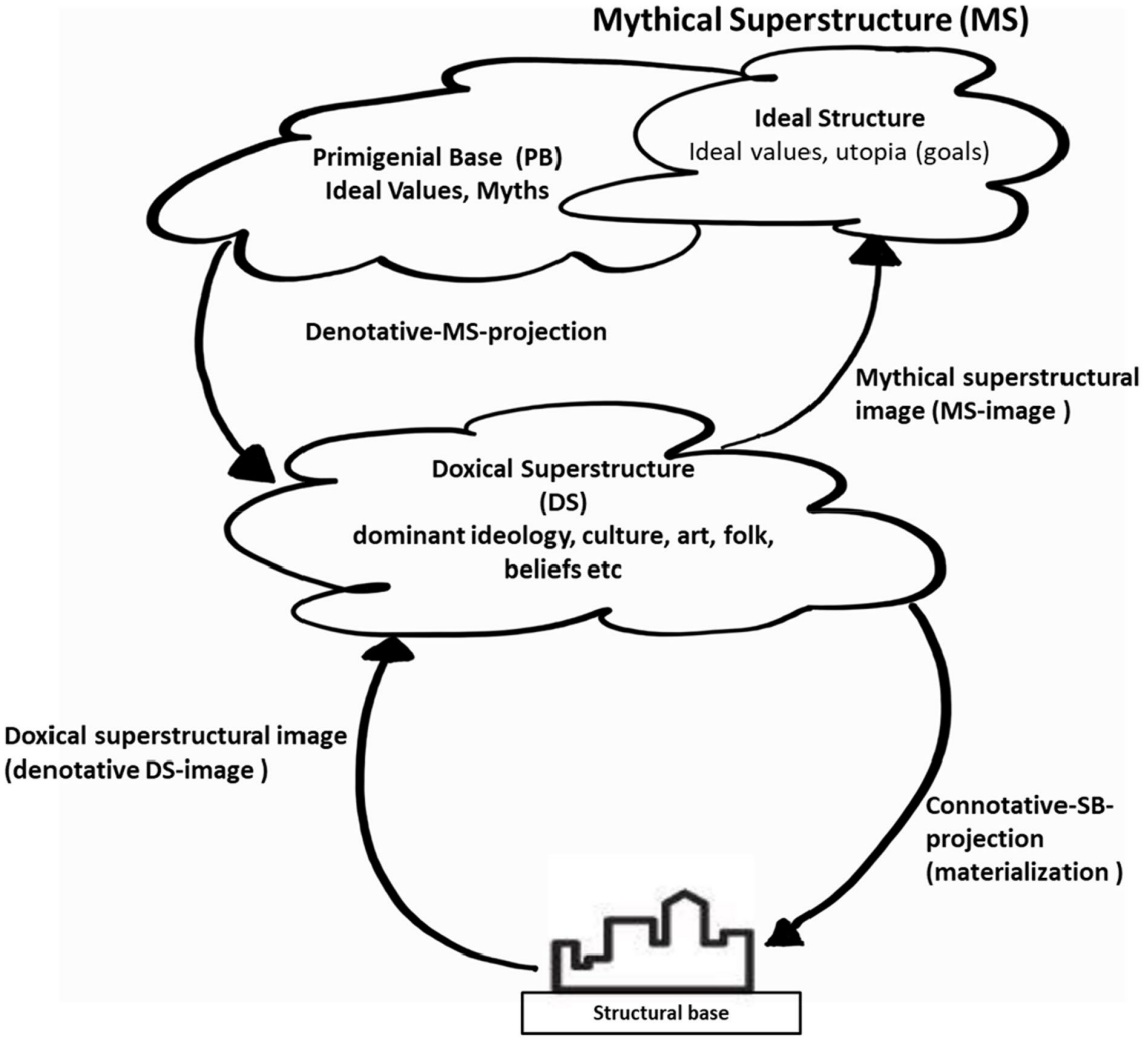
Structural base and superstructures (Nescolarde-Selva and Usó-Doménech, 2014, p. 69).

Epistemic norms play a central role in developing and upholding social and cultural logic. In their study on language, myths, and complex ideologies, Nescolarde-Selva and Usó-Doménech (2014) conceptualised successive systems that play varying roles in carrying out ideological objectives and offered useful thinking that can be leveraged in an examination of coloniality and the *doxic*—that is more broadly understood as that which is taken for granted and viewed as normal and natural in society. It appears as part of the natural social order, although socially organised and thus unnatural or doxic. Nonetheless, that which is doxic constitutes what society may broadly understand as accepted assumptions, including that which is epistemic and guides societal norms, culture, language, and the framing and conceptualising of ways of being. It extends to the framing of discourse on being human as praxis and the borders that govern the discourse to create disciplines. For example, the epistemic assumptions that guide our discourse on the economy, levy assumptions built since Adam Smith to abstract ways of doing. These then contributed to discourse and the building of narratives that then transformed into a discipline. In turn, the discipline has had a bearing on the praxis of being human through a policy that organises people based on epistemic assumptions about abstracted forms of doing, such as manufacturing and productivity. Recognising the construct of the social mechanisms at play requires the capacity to identify and conceptualise social phenomena. It also requires the capacity to break down the undifferentiated whole into discrete components and attach nomenclature to the varying parts that constitute the social order (Figure 2). This is important for our understanding of the “coloniality of knowledge and of being” (Mignolo and Walsh, 2018, p. 136).

The doxical superstructure constitutes “dominant ideology, culture, science, art, folk, belief, etc.” (Nescolarde-Selva and Usó-Doménech, 2014, p. 69). Beneath the superstructure lies the structural base, wherein the context of this study, I would argue, one can find the onset of the current global order; the structural base can be understood as the instigator on which all the ideas in the doxical superstructure are broadly based. The structural base comprises sub-structures and the key events from which the superstructure emerged.

Following this line of thinking and adopting Beetham’s (1991) conceptualisation of the legitimation of power, with specific reference to the notion of conformity to established rules, one may look for tensions and slippages in the establishment of rules such as in international law by excavating the imperial logic on which the rules were built. That is to say, the process of empire-building shaped the practice and discipline of international law and governance, as previously discussed in the case of Hugo Grotius, the Dutch-East India company, and *Mare Liberum* (Krishna, 2001). This colonial impact on international global law and norms is visible elsewhere such that Grovogui (1996) contends “that the modern law of nations has been proposed by a select group of nations, not as the ethical basis of a universal order, but as a means to hegemony…enabling Europe to undermine the other’s subjectivity and sovereignty in the international order.” Building on Grovogui’s (1996, p. 43) assertions, there currently exists a body of research that looks to delegitimise global order (Krishna 2001; Mignolo and Walsh, 2018; Ndlovu-Gatsheni, 2018).

## Resistance to coloniality

Using Beetham’s (1991) research on legitimation, the intellectual tools of justification from the university can be argued to be legitimations for the conscience of the powerful as well as tools to secure consent from the subordinates to avoid subversion. I would argue that coloniality is experiencing as elaborate a ruse as slavery and early capitalism experienced, in terms of the theories of justification that varied from Aristotelian conceptions of slave nature to racial theories of inferiority to appease the conscience of the powerful. Coloniality is, at present, as logically impossible as the relegation of slaves and early industrial Western workers to the category of objects (Beetham, 1991), although workers in some parts of the world are still treated as badly as early industrial workers in the West, with some experiencing modern-day slavery across the globe. I contend that this is in keeping with the morally deficient principles and norms established within the structural base. This argument is not to minimise the role or the agency of governments and populations in the global south. Instead, it is to highlight that there are structural barriers to fighting against this most elaborate ruse and that these barriers are in part underpinned by a race-based moral relativism that maintains the “grounds for compliance” despite clearly unequal power relations, whether it is in global order or knowledge production (Beetham, 1991, p. 34). These challenges are attached to the manner in which the structural base evolved, which is to say, the colonial did not exist without the anticolonial, although the initial resistance to colonialism did not win; it existed, and in this way, it shaped the structural base. For example, resistance to colonialisation shaped how the colonial administration operated across colonies and shifted ways of being for the coloniser and by extension how whiteness materialised then (Chibber, 2017). In these ways, it in part informed the doxical superstructure and how components of it materialise in the contemporary, for example, the role of the university in producing intellectual tools that form justification grounds for international governance—including inequity in global governance and power asymmetries. These tools are then definitive of how coloniality and whiteness materialise in their exertion, conservation, and propagation of power in the present and become a reference point for legitimacy.

**Figure 3:**
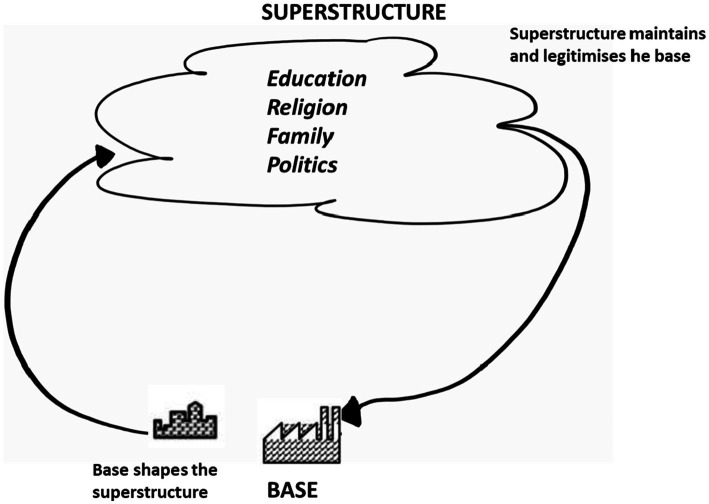
Superstructure and base legitimisation (Nescolarde-Selva and Usó-Doménech, 2014, p. 69).

Compartmentalisation within the structural base and superstructure, importantly, facilitates the naming of discrete components and, successively, the interrogation of the global order and its underpinning mechanisms which are “androcentric, hetero-patriarchal, racist, sexist, Euro-North American-centric…colonial, and capitalist hierarchies and heterarchies” (Ndlovu-Gatsheni and Ndlovu, 2021, p. 90). Nescolarde-Selva and Usó-Doménech (2014, p. 63) assert that ‘not to distinguish between complex ideologies, and ordinary ideas, beliefs, myths, prejudices and so forth, is to lose a whole level of analysis.” One may argue that, in terms of stratifying power, it is not just a matter of distinguishing between “complex ideologies and ordinary beliefs” (Nescolarde-Selva and UsóDoménech, 2014, p. 63). Rather, it is a matter of also bringing both ideology and prejudice to understand how one is associated with the other and to understand how ideology can act as the driving force behind prejudice. In this way, one can engage with examining the embedded power at each level. If power is to be understood as “the production of intended effects,” (Russell, 1938, p. 18) or one’s “ability to produce intended effects upon the world around them” (Beetham, 1991,p. 43), then distinguishing between low-level ideas that are not immaterial but yield less power and, for example, state-sanctioned ideologies becomes important. Doing so facilitates the examination of every power (Bourdieu and Passeron, 1990), including the power that conceived society epistemically, the power that articulated it ideologically, the power that ordered it practically, and the power that upholds the status quo. It is not without merit to argue that scholars such as Robinson (2000) in their articulation of racialised capitalism do exactly that. Robisnson’s (2000) *Black Marxism* centralises the racial struggle against the backdrop of the formation of the present modern world encapsulating the conceiving epistemology and guiding ideology and operational practicalities, and it is in this stratification that one can examine every power. That is “every power which manages to impose meanings and to impose them as legitimate by concealing the power relations which are the basis of its force, [adding] its own specifically symbolic force to those power relations” (Bourdieu and Passeron’s, 1990, p. 4). It is in this stratification and process of highlighting the loci where power resides that one can engage critically with the process of denaturalising the doxic and understanding the processes that have legitimised and continue to legitimise power. Whilst this creates room for a more nuanced conceptualisation of power, it is also a more complex conceptualisation of power. On one level, it considers how the superstructure, of which education (read university) is a part of, exercises power in its legitimisation of the structural base (read coloniality) (See Figure 3).

**Figure 4:**
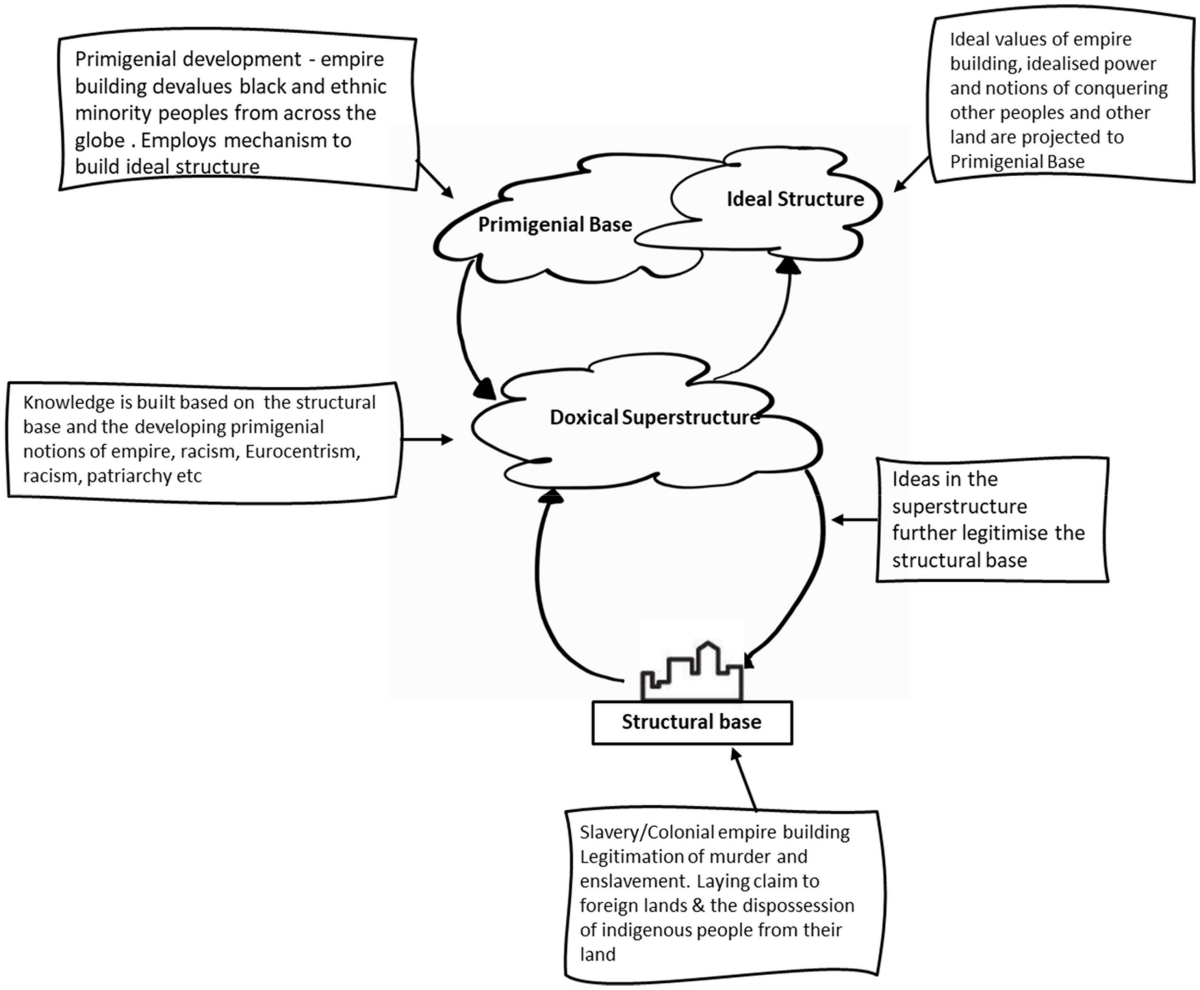
Evolving colonial social logic [adapted from Nescolarde-Selva and Usó-Doménech (2014), p. 69].

Universities offer us the opportunity to take snapshots of the doxical superstructure in context and are rich grounds for multi-lens analysis. They enable us to go backwards to map the genesis and evolution of the superstructure and the ideas that are embedded within. For example, they enable us to map the emergence of a discipline and steep it in its historical context to understand where, why, and who was involved in its emergence, as well as consider how the discipline has further legitimised the base and the mechanisms through which it will continue to legitimise the structural base (see Figure 4).

Figure 4 illustrates imperial ideological continuation in the university, showing that the question of race and coloniality is embedded in the structural base, shaping the superstructure and affirming both. The affirmation in the superstructure materialises in global norms and laws, as well as in educational institutions of which universities are a critical aspect.

## What of the university and the neoliberal social imaginary?

I have demonstrated that the university is part of what legitimises the structural base. It is also the place where theory is conceptualised, constituting worlds—in Figure 1, this would be the mythical superstructure where theories that constitute ideals are made. In the current contemporary context, these ideals are fashioned around a neoliberalism that has its roots in the structural base and projects its values in the ideal structure. These values are not only associated with economic theory but extend to the cultural imaginary where representations of its subjects are created and identity formation on life and death is mobilised. They set forth a basis for differentiation between important and unimportant lives and provide a global economic structure that functions to reinforce that differentiation (Ibled, 2020). The challenge for the university is in its function as a producer of theory and whether those theories are predominantly informed by an illegitimate, racist structural base and the subsequent impact of that on how society functions. To further interrogate this, it is necessary to take a closer look at the university’s role in society and the function of theory within society. An exemplar of the power of theory would be the function of economic abstraction in society. The emergence and function of theory can be understood in three parts for analysis.

1. Theory is always a distinct theory of the world, underpinned by the social and cultural logic from which it emerges.
2. The power of abstractions and influence of abstractions are bound to the abstraction’s “capacity to constitute a world” and, to a lesser extent, its accuracy (Mbembe, 2021, p. 19).
3. Theory flows between abstraction and action and, in this back-and-forth process, it formulates guidelines that operate across a continuum from undesirable to idealised.

The flow that exists between sociocultural logic, abstraction, and action can be viewed as an interplay between the act of theorising and society. Theory in itself is not value-free, and in the move from theory to action, theory can embed its constituent parts into society, where knowledge and values are relayed. Following this line of thinking, one can interrogate Eurocentrism within the university and the university’s role in society. Using Amin’s (2009, p.154) definition of Eurocentrism, one can view it as “a theory of world history” that centralises Europe in human history, “which is a cultural expression of Euromodernity, mediated by the inferiorization of others and the superiorization of Europeans” (Ndlovu-Gatsheni and Ndlovu, 2021, p.2). It is fundamentally grounded in epistemic violence. The question then becomes, does the university perpetuate epistemic violence? The growing body of literature that is in pursuit of epistemic justice, against the backdrop of the socioeconomic and political challenges that are acute within former colonies, indicates the urgent need to put the university on the stand. It also indicates the need to pose questions on the interplay that exists between the maintenance of colonial global power structures—coloniality and knowledge—as well as the need to interrogate its role in epistemic governance and exclusionary praxis and how that has both historically and in the present disadvantaged and contributed to the oppression of the “restern world” (Ndlovu-Gatsheni and Ndlovu, 2021, p. 89).

Decolonisation’s fundamental task was to take hold of the state. Decoloniality’s task to decolonise knowledge is arduous and nebulous, and it raises a litany of questions. The weapon of protest in the colonial era was to build national liberation armies, take up arms, drive out the invaders, and take the state (Mignolo and Walsh, 2018). What of decoloniality’s weapon of protest and the cognitive equivalent of national liberation armies that can destabilise the very foundation of the cognitive colonialism that is the universality of Western epistemology? I put forward the argument that if the university wanted to begin to transform itself, moving away from coloniality, it is mere moments away from the starting line because there is banality in my argument. The question of how has been addressed either directly or implicitly by the likes of de Sousa Santos (2017) in his comprehensive work, Decolonising the University, and other decolonial scholars (such as Ake, 1982; Mudimbe, 1988 Maldonado-Torres, 2016; Bhambra et al., 2018; Ndlovu-Gatsheni, 2018, etc.). Decentralising Western epistemology, therefore, requires critical engagement with what we know of imperialists and colonialists in terms of the perverse logic that sought to destroy, disfigure, and distort the knowledge systems of those it sought to oppress and how that has translated into the university currently (Fanon, 2001). Colonial logic instigated a process of dismemberment and defamiliarizing of other ways of knowing to plant European memory in pursuit of universalising a Western epistemology that is bound to ideals that constitute the mythical superstructure (see Figures 1, 4). It erected boundaries to impose colonial conceptual frames and borders that would mark the end of ways of knowing and silence the global majority who now sits on the periphery of disciplines (Mignolo and Walsh, 2018, p. 135). This necessitates the adoption of methods such as Capers (2006) *Reading Back, Reading Black*, across the disciplines, to continuously ask who is disempowered and dispossessed.

## Of borders and frontiers

The task at hand is not merely a question of marked boundaries within the disciplines, although the question of boundaries is important in decolonial thinking because it facilitates the re-valuation of the lines that mark the frontiers of where disciplines start and end. To be able to engage with the question of re-valuating disciplines, the superseding question must not be discipline bound but rather bound to contextualising and provincializing Western knowledge, rationalities, and logic (Ndlovu-Gatsheni, 2018), that is, to move beyond the limits of Western epistemology and the colonial idea of universality and totality of knowledge (Mignolo and Walsh, 2018). It should broach instead on the idea of borderlines that mark the end of regional knowledge that is bound to locality, not universality. In this way, room for a pluriversal approach to knowledge is created across the university. de Sousa Santos (2014) argues this to be imperative currently because the West has begun to show signs of exhaustion. Using political science as an example, de Sousa Santos notes the shift in critical theory from the formulation of nouns, such as socialism or liberalism, to the era of impotence and exhaustion of ideas that has ushered in the age of adjectives, such as “neo” liberalism or “sustainable” development (de Sousa Santos, 2014). The call to move toward pluriversality for decolonial scholars is commensurate with Lorde’s (2018) assertion that “the master’s tools will never dismantle the master’s house.” For what would it mean for the tools of racialised capitalism to examine the fruits of the same racialised capitalism? “How do you decolonise political theory if you do not open up your way of thinking to forms of governance beyond the nation-state?” ask Mignolo and Walsh (2018, p. 136). There is, therefore, a need to change the terms of the conversation to centralise what matters most, that is, knowledge rather than discipline (Mignolo and Walsh, 2018). Where disciplines provide spheres that are useful for thinking and theorising, being human as praxis is an entangled constellation of interweaving and interlocking praxical spheres. (Ndlovu-Gatsheni and Ndlovu, 2021, p. 91). Therefore what is important for decolonial thinking is the “knowledge weaved around concepts such as politics and economics...[rather than] politics and economics as transcendental identities” (Mignolo and Walsh, 2018, p. 136). It is the knowledge that speaks to being human as praxis that has been or is being silenced, which can deconstruct the artificial frontiers that uphold widely accepted norms and assumptions within disciplines that are bound to the colonial matrix of power. Thus, liberating knowledge must start with changing the terms of conversations that form its basis, shifting from the questions within the boundaries that regulate the discipline to the frontiers of the epistemic assumptions on which they are built.

## Teaching global order

Exploring what the university teaches and how the university teaches disciplines that concern themselves with global order and the world system, such as development, international relations, and global political economy, make for a good starting point that can benchmark the level of critical engagement with decoloniality within the university. These subjects give rise to the stark tensions in the colonial and anticolonial that are important for negotiating the institutional implications of decoloniality for the university. Within the disciplines of international relations and development, the elephant in the room remains, that is, development and international relations’ organisational and institutional architecture are imbued with coloniality (Rutazibwa, 2018). Invisible as they may be, the colonial matrices of power are made evident in the global imperial design that has maintained the colour line, which separates the colonised and coloniser. These disciplines exist within long-established boundaries that fit within the scope of imperialistic endeavours. They demonstrate a deep rooting in the structural base and effectively affirm it through theorisation and the creation of concepts and methodologies that guide knowledge production and bend the arc of global order toward the West, to uphold an unequal and racist global ordering that preaches a fallacy of philanthropy (Gomberg, 2002) and remains broadly Eurocentric in its discourse. Through ‘development’, they endeavour to universalise Western conceptions of human praxis at the cost of cultures and philosophies of those who exist beyond the West, to sustain a dominant/subordinate power system that is defined by the superiority of the Global North and the inferiority of the Global South. This is evidenced by the silencing and absence of Global South scholars on curricula, which has instigated student and teacher-led decolonial movements that have fought against the dearth of critical approaches to reading texts that have imperial and racist underpinnings and continue to sanitise the crimes of coloniality (Tegama and Fox, 2023). In these ways, the university can be “complicit in reproducing, invisibilising and legitimizing” and normalising the colonial project, the current global relations and the violence that maintains them and constitutes the doxic (Rutazibwa, 2018, p. 160). It can do so to impose epistemic and psychological violence on Black and Ethnic Minority (BAME) students who undertake courses that silence their lived experiences and histories, imposing feelings of unbelonging amongst the BAME student bodies (Shieber, 2020).

## Conclusion

I have predominantly engaged in critiquing the university and the drawing of lines that mark the continuity of imperial ideology to facilitate a broad understanding of the need to delegitimise and decolonise systems and scholarship. I engaged with Beetham’s (1991) conception of the legitimation of power and triangulated it with a conceptualisation of the doxic and structures that legitimise and normalise. I have done so to situate the university in a global context, interrogate its function, and demonstrate that abstractions and theorisations are never devoid of power or social and cultural logic. Although they are useful analytical devices that frame knowledge and draw boundaries that bring certain realities into focus, they always simultaneously leave others out of the picture. “Every effort at knowledge is ineffably accompanied by a simultaneous and unavoidable concealment” (Krishna, 2001, p. 404). This necessitates the adoption of critical and oppositional reading methods such as Capers (2006) *Reading Back, Reading Black*. To mark the points where power lies, find tensions, and investigate who is silenced, one must take note of where and why they are silenced and open the door to investigating the underpinning logic. This is not only true of historical works but also of present-day theorists who are intimately engaged with coloniality. It is, therefore, important for the university to move beyond the neoliberal social imaginary to adopt a critical approach to its role in the superstructure and strategically exercise intentionality in what it chooses to legitimise or delegitimise within the structural base.

Whether we view the university as a colonial agent or an innocent bystander has important implications for society at large. If coloniality is the conceptual apparatus that propagates the “racial, political economic, social, epistemological, linguistic and gendered hierarchical orders imposed by European colonialism that transcended ‘decolonisation’ and [continues] to oppress in accordance with the needs of pan-capital (i.e., economic and cultural/symbolic) accumulation” (Davies, 2019, n.np), then decoloniality in the university lies in moving scholarly discourse beyond the parameters of the neoliberal social imaginary and beyond short-circuiting discourse to, for example, deprive students of the option to critically engage with large-scale egalitarian systems or capitalism’s ongoing moral failures. In short-circuiting the curriculum, the university short-changes both its students and society at large by fostering political quietism. Drawing students away from context and the reality of racialised social relations in the classroom whilst they experience them in their everyday social relations deprives them of the capacity to make sense of and effectively come up with solutions to tackle not only the global challenges of their time but also the challenges at home, in the cities, and communities.

For the decolonial scholar(s) who may find themselves in institutions that are resistant to change, small ruptures are necessary. In, for example, reshaping approaches to teaching the curriculum by adopting critical and oppositional methods and pluriversal approaches to teaching. It is necessary to steep theories in historical and social contexts from which they emerge and equip students with the methods and confidence to deconstruct racialised capitalism’s suppositions on progress, human nature, and racism as organising principles of global order. It is through these small ruptures that we can collectively destabilise coloniality and prove its legitimation of power within the university, in society, and in global norms to be based on shaky moral grounds.

## Author contributions

The author confirms being the sole contributor of this work and has approved it for publication.
